# A Record of the Department of Health, New York

**Published:** 1918-09-21

**Authors:** 


### THE CORRELATION OF RESEARCH.

### A Record of the Department of Health, New York.

In the two volumes* before us are recorded some of the more notable advances in medical and sanitary knowledge, for the years 1912-15, made by private investigators and clinicians and by workers in the Health Laboratories of the State of New York. The value of such a publication is more remote than immediate, and its interest naturally greater for the research student than for the active practitioner of medicine. But that its publication should be deemed worth while is a striking tribute to the just estimate made by our Allies of the vital importance of keeping all scientific workers in intimate touch with the advances made by their *confrères* on similar lines; and of the immense significance of abundant statistics in the investigation of the problems of epidemic disease. Medical statistics are matters not to be lightly taken in hand, and the scientific training of the student of medicine on this side of the water, however admirable in some ways, certainly affords him little or no help in this respect. Although most of the articles are of a severely technical nature and as such of but slight interest to the general medical reader, a section in each year is devoted to applied therapeutics and to clinical studies of cases with the accompanying laboratory findings, in which valuable light is thrown upon the etiology of commonly occurring diseases and the value of new-fangled treatments established.

Thus in 1913 we find an investigation upon the value of serum treatment in pneumonia, the results being inconclusive. In 1914 a similar investigation was conducted into the value of vaccine prophylaxis in an epidemic of pertussis in the Hebrew Infant Asylum, N.Y., and in the same hospital the injection of blood from an immune patient was used to check an epidemic of mumps. The value of the pertussis vaccine in checking the epidemic seems to have been very questionable, for a large number of those who were thus treated developed the disease; and the vaccine seems to have had little or no effect in checking it when once established. As an example of American thoroughness in questions of infant welfare may be mentioned the establishment of a special whooping-cough clinic in New York. "The facilities for the rational treatment of pertussis have not been any too numerous in New York," the report says. "In the vast majority of the outpatients' departments the whooper is looked upon as a pariah and treated accordingly. Thus an anomalous situation, a kind of vicious circle, is established for the sick poor. Applying to the dispensary for treatment, the mother is told to go home without delay and keep the child indoors until the whoop stops; at the same time she is given a card with printed instructions enjoining her to have the child out in the open air as much as possible. When the paroxysms become alarming, the lodge physician [*i.e.* the panel doctor] is sent for and the overworked contract practitioner is only too glad to diagnose whooping-cough and advise the family not to call him again because, as they well know, there is no cure for the disease." How true is this of London or any other big centre, and how far are our own health authorities from adopting any such simple solution! In less than five months 376 cases were treated, and a large number such as this rendered possible a number of important investigations. Thus the complement fixation test for the Bordet-Gengou bacillus was not found to be of any practical value, since it only occurred in some 50 per cent. of cases, and that not until the fourth day, when, of course, the whoop was well developed. Nor was the typical organism isolated with any constancy, its presence clinching the diagnosis in a doubtful case, but not disproving it if the bacteriological result was negative. That the organism is rarely found later than the first week of the paroxysmal stage seems, however, to indicate that the early part of the disease is the most infectious, and that there is no necessity to confine a child to the house after the whoop has been established for a week. Similar conclusions to those recorded above as to the value of the vaccine were arrived at in the Hebrew Infants' Hospital.

The value of these extended observations and their reflex upon the private practitioner cannot be overestimated. How often is a physician tempted to recommend some much-lauded vaccine preparation or drug which he has perhaps seen advertised in the medical press, and how often does he ascribe its non-success to some mistakes on his own part rather than to the inefficacy of the preparation! In this connection may be mentioned an investigation on commercial preparations of the *Bacillus Bulgaricus*, a treatment much in vogue a few years back for certain intestinal ailments. The majority of preparations sold were, it was found, sterile by the time they reached the consumer, and the remainder contained only a small fraction of the bacilli represented by the manufacturers. Systematic inquiries of this kind would do much to prevent the foisting of fraudulent articles on the market. Mention must be made of the admirable papers by Dr. Alfred F. Hess, M.D., on infantile disorders, and of the painstaking bacteriological examinations of certain lady investigators.

With commendable but tantalising reticence the Department of Health refrains from giving any particulars of the number and constitution of its staff, or the salaries paid to them. Dare we assume that in the United States the research worker is deemed worthy of his hire, and that there science is at least as lucrative an employment as shop-keeping?

* *Collected Studies from the Bureau of Laboratories—Department of Health, City of New York.* Vols. vii and viii.

